# Comparable Infection Risk of Ocrelizumab and Rituximab in Multiple Sclerosis in a Nationwide Swedish Cohort Study

**DOI:** 10.1002/ana.78286

**Published:** 2026-06-17

**Authors:** Thomas Frisell, Sebastian Hedberg, Fredrik Piehl

**Affiliations:** ^1^ Division of Clinical Epidemiology, Department of Medicine Solna Karolinska Institutet Stockholm Sweden; ^2^ Department of Clinical Neuroscience Karolinska Institutet Stockholm Sweden; ^3^ Academic Specialist Clinic, SLSO and Department of Neurology Karolinska University Hospital Stockholm Sweden

## Abstract

In multiple sclerosis (MS), anti‐CD20 therapies have been associated with increased infection risk, but whether this risk differs between rituximab and ocrelizumab is not known. Using a nationwide cohort, we studied infection risk and relapse outcomes among 16,872 treatment episodes with rituximab, ocrelizumab, or other disease‐modifying therapies (DMTs) between 2014 and 2025. Infection outcomes included serious infections, hospital‐diagnosed infections, and antibiotic use, and relapse risk was assessed as a marker of efficacy. Both rituximab and ocrelizumab were associated with higher infection rates than other DMTs, while relapse rates were substantially lower. Infection and relapse risks were similar between rituximab and ocrelizumab. ANN NEUROL 2026;100:788–792

Multiple sclerosis (MS) is a chronic disease that often requires long‐term treatment to reduce the risk of accumulating neurological disability. Compared with other disease‐modifying therapies (DMTs), anti‐CD20 therapies, also known as B‐cell–depleting therapies (BCDTs), provide high efficacy in suppressing inflammatory disease activity and are generally well tolerated, resulting in high treatment persistence.^1^ Their main disadvantage is an increased risk of infections, although this was not clearly evident in pivotal trials of ocrelizumab and ofatumumab,^2^, ^3^, ^4^ but with 3 infection‐related deaths reported in the phase 3 ublituximab trial.^5^ Longer‐term follow‐up in population‐based cohorts shows that rituximab exposure is associated with an approximately 2‐fold increased risk of infection‐related hospitalization.^6^, ^7^ Evidence for clinically meaningful differences in infection risk between individual BCDTs, however, remains limited.

A recent meta‐analysis suggested that ocrelizumab was associated with numerically higher counts of pulmonary, urinary tract, and gastrointestinal infections among BCDTs, followed by rituximab and ofatumumab.^8^ Substantial heterogeneity in study designs, patient selection, and outcome ascertainment means that reported findings should be interpreted with caution. More recently, Cerono and colleagues^9^ reported markedly higher adjusted risks of hospitalization and infection‐related hospitalization with rituximab compared with ocrelizumab in 2 retrospective cohorts from California.

Rituximab has become a widely used off‐label alternative to approved MS therapies in several countries and is included on the World Health Organization's Model List of Essential Medicines, largely because of its substantially lower cost.^10^ If rituximab is associated with a substantially higher infection risk than ocrelizumab, the economic benefits of its use could be offset by increased morbidity.^9^

To address this question, we conducted a nationwide cohort study using Swedish administrative health registers and the Swedish MS Registry to compare infection‐related hospitalizations, antibiotic prescriptions, and relapse rates among people with MS treated with rituximab or ocrelizumab.

## Methods

We included all patients recorded in the Swedish MS register starting any DMT between January 1, 2014 and December 31, 2024, with follow‐up to December 31, 2025. In addition to rituximab and ocrelizumab, we defined a third group “other DMTs” as any of cladribine, dimethyl fumarate, fingolimod, natalizumab, and teriflunomide. Only first ever DMT start in each group was included, and each subject could, therefore, enter the analysis more than once, but only once for each DMT‐group. The study was approved by the Swedish Ethical Review Authority (dnr. 2021‐02384).

As described previously,^7^ data on infection outcomes and potential confounders were extracted through linkage to several of Sweden's high‐quality national census and healthcare registers. Three definitions of infections were used: (1) serious infections defined as hospitalization because of infection as main diagnosis, (2) any recorded main or contributory diagnosis of infection at hospital visit, including outpatient/ambulatory care, and (3) any filled prescription of antibiotics. Additionally, time to first relapse was included as a fourth outcome to provide data on the relative efficacy of the treatments. Confounders included demographic factors, MS‐specific disability and clinical scales, comorbidities, and general health. For full definitions, see Tables S1 and S2.

Patients were followed from DMT initiation until the first event, with separate analyses performed for each event type, or until censoring due to death, emigration from Sweden, DMT discontinuation, or December 31, 2025. Patient characteristics at DMT start was tabulated by DMT group and incidence rates of each event calculated per 1,000 person‐years at risk. Hazard ratios (HRs) between DMT groups were estimated in univariable and multivariable Cox regression, with time since DMT start as time scale. Fully adjusted model included all tabulated patient characteristics, with age modeled as third‐degree polynomial, and clinical scales with non‐negligible missing data (Expanded Disability Status Scale, Symbol Digit Modalities Test, and MS impact scale‐29) as 4 categories: tertiles plus a missing data indicator. The sandwich estimator was used (clustered on person id) to correct confidence intervals for inclusion of same patient in different DMT groups. Sensitivity analyses were (1) restricted to relapsing–remitting MS (RRMS), (2) excluding coronavirus disease 2019 (COVID‐19) from serious infection definition, and (3) alternative covariate adjustment using propensity score weighting.

## Results

We included 9,704 treatment starts of rituximab, 148 of ocrelizumab, and 7,020 of other DMTs (cladribine: 285, dimethyl fumarate: 3,189, fingolimod: 726, natalizumab: 2,125, and teriflunomide: 695). The average follow‐up time before censoring was 5.4 years on rituximab, 4.7 years on ocrelizumab, and lower in the other DMT group (3.6 years), reflecting higher DMT discontinuation rates (Table S3).

Despite ocrelizumab being a much less frequent choice than rituximab, the patients starting each BCDT were overall similar and differed from those starting other DMTs in several respects (Table 1). BCDT was less often used as the first ever DMT and more frequently used in patients with higher MS associated disability and progressive disease course. Patients starting rituximab were also on average a bit older. Although a majority of those starting ocrelizumab had previously used rituximab (n = 91, 61%), none had used ocrelizumab before rituximab, suggesting that ocrelizumab may have been preferentially used in patients switching from rituximab or in the context of clinical trials.

**TABLE 1:** Patient Characteristics at DMT Start

	Rituximab	Ocrelizumab	Other DMT
N	9,704	148	7,020
Yr of DMT start	2018 (5)	2020 (3.4)	2016 (5.2)
Age, yr	41.6 (12.1)	37.9 (11.0)	38.6 (11.3)
F	6,702 (69.1%)	100 (67.6%)	4,991 (71.1%)
Immigrant	1,630 (16.8%)	37 (25.0%)	1,131 (16.1%)
Education <12 yr	5,252 (54.1%)	76 (51.4%)	3,676 (52.4%)
MS course			
RRMS	8,121 (83.7%)	128 (86.5%)	6,657 (94.8%)
SPMS	1,078 (11.1%)	10 (6.8%)	252 (3.6%)
PPMS	466 (4.8%)	10 (6.8%)	87 (1.2%)
Yr since diagnosis	6.2 (7.4)	6.5 (6.6)	4.5 (6.3)
Prior DMTs			
None	3,275 (33.7%)	30 (20.3%)	3,401 (48.4%)
1	2,680 (27.6%)	37 (25.0%)	1,916 (27.3%)
≥2	3,749 (38.6%)	81 (54.7%)	1,703 (24.3%)
Any relapse last yr	1,913 (19.7%)	32 (21.6%)	1,716 (24.4%)
EDSS	2.4 (1.9)	2.3 (1.9)	1.9 (1.5)
MSIS‐29 physical	24.5 (22.8)	28.5 (24.5)	20.5 (20.7)
MSIS‐29 psychological	33.2 (24.0)	39.3 (24.9)	31.4 (23.9)
SDMT	51.8 (14.0)	53.7 (11.6)	52.2 (12.4)
Medical history, last 5 years			
Serious infection	435 (4.5%)	9 (6.1%)	281 (4.0%)
Infection	1,976 (20.4%)	34 (23.0%)	1,296 (18.5%)
Antibiotics	5,806 (59.8%)	107 (72.3%)	4,192 (59.7%)
MACE	123 (1.3%)	3 (2.0%)	86 (1.2%)
Arrhythmia	135 (1.4%)	0 (0%)	106 (1.5%)
Hypertension	1,715 (17.7%)	26 (17.6%)	1,013 (14.4%)
Antidepressants	2,644 (27.2%)	33 (22.3%)	1,679 (23.9%)
Malignancy	122 (1.3%)	0 (0%)	82 (1.2%)
Diabetes	364 (3.8%)	3 (2.0%)	192 (2.7%)

All numbers are mean (SD) or n (%).

DMT = disease modifying treatment; EDSS = Expanded Disability Status Scale; F = female; MACE = major adverse cardiovascular event; MS = multiple sclerosis; MSIS‐29 = MS Impact Scale‐29; PPMS = primary progressive MS; RRMS = relapsing–remitting MS; SD = standard deviation; SDMT = Symbol Digit Modalities Test; SPMS = secondary progressive MS; yr = year.

The crude rate of serious infections was 22 and 30 per 1,000 person‐years on rituximab and ocrelizumab, respectively, representing a statistically significant and more than doubled rate compared to other DMTs (Table 2, individual DMTs in Table S4). The relative risk was equally significant, although somewhat smaller for the broader infection definition, which may be expected given its higher base rate. For antibiotics use, which was approximately 10 times as frequent as events of serious infections, the adjusted rate was increased by 26% on rituximab and 73% on ocrelizumab (adjusted HR on ocrelizumab vs rituximab 1.37, 95% CI: 1.10–1.71). In contrast, the relapse rate was substantially lower (more than halved) on BCDT than other DMTs, but similar between rituximab and ocrelizumab (adjusted HR ocrelizumab vs rituximab 0.96, 95% CI: 0.40–2.32).

**TABLE 2:** Incidence Rates and Crude and aHRs from Cox Regression by DMT

	PY	Events	IR per 1,000 PYs	HR (95% CI)	aHR (95% CI) ref: other DMT	aHR (95% CI) ref: rituximab
Serious infection						
Rituximab	48,575.2	1,048	22	2.56 (2.21–2.98)	**2.16** (1.82–2.57)	1.0 (ref)
Ocrelizumab	632.2	19	30	3.60 (2.24–5.77)	**2.92** (1.72–4.98)	1.35 (0.81–2.25)
Other	24,283.9	215	9	1.0 (ref)	1.0 (ref)	**0.46** (0.39–0.55)
Infection						
Rituximab	42,950.1	2,779	65	1.73 (1.59–1.87)	**1.69** (1.53–1.86)	1.0 (ref)
Ocrelizumab	555.5	52	94	2.42 (1.83–3.21)	**2.18** (1.59–3.01)	1.29 (0.95–1.76)
Other	22,296.5	812	36	1.0 (ref)	1.0 (ref)	**0.59** (0.54–0.65)
Antibiotics use						
Rituximab	27,045.5	6,016	222	1.33 (1.27–1.39)	**1.26** (1.18–1.34)	1.0 (ref)
Ocrelizumab	324.8	100	308	1.84 (1.50–2.25)	**1.73** (1.38–2.17)	**1.37** (1.10–1.71)
Other	14,917.3	2,709	182	1.0 (ref)	1.0 (ref)	**0.79** (0.74–0.85)
Relapses						
Rituximab	50,058.8	413	8	0.46 (0.40–0.52)	**0.38** (0.32–0.44)	1.0 (ref)
Ocrelizumab	670.5	6	9	0.55 (0.24–1.23)	**0.36** (0.15–0.88)	0.96 (0.40–2.32)
Other	23,829.9	567	24	1.0 (ref)	1.0 (ref)	**2.65** (2.25–3.10)

Hazard ratio adjusted for age, sex, immigrant status, education level, MS course, number of previous DMTs, time since diagnosis, having experience relapse in last year, EDSS, MSIS‐29, SDMT, and recent history of other disease, including infections, in the last 5 years. The 2 aHR columns differ only by choice of reference group. Significant results in bold.

aHR = adjusted hazard ratio; CI = confidence interval; DMT = disease modifying treatment; EDSS = Expanded Disability Status Scale; aHR = adjusted hazard ratio; IR = incidence rate; MS = multiple sclerosis; MSIS‐29 = MS Impact Scale‐29; PYs = person‐years; SDMT = Symbol Digit Modalities Test.

The differences in infection rate between BCDT and other DMTs were not explained by the observed differences in patient characteristics. Although adjustment for these attenuated the HRs for serious infection somewhat for both rituximab and ocrelizumab, and slightly also for other hospital‐diagnosed infections, most of the association remained and there was no impact of the adjustments in the most well powered analysis of antibiotics use. Differences also remained virtually unchanged in sensitivity analysis restricted to patients with RRMS at DMT start, when excluding COVID‐19; when (at reviewer request) adjusting for covariates through propensity score weighting; and when restricted to first ever DMT start (Tables S5–S8).

## Discussion

In this large nationwide register‐based cohort study of people with MS followed over several years, we confirm previous findings of increased infection rates with BCDT compared with other contemporary DMTs,^6^ which also have shown no clear influence of prior DMT exposure or duration of BCDT treatment.^7^ This does not exclude that cumulative immunosuppressive exposure and treatment duration remain biologically plausible contributors to infection risk and may differ across cohorts, potentially interacting with factors such as age, disability level, progressive phenotype, and hypogammaglobulinemia.^8^

In relation to the primary objective of the present study, we found that differences between rituximab and ocrelizumab were small and largely non‐significant. This comparison is limited by low statistical power because of the smaller size of the ocrelizumab group, particularly for rarer events. Nonetheless, antibiotic use, the most frequent infectious outcome, was nominally higher with ocrelizumab than with rituximab. These findings contrast with a recent report by Cerono and colleagues,^9^ who observed several‐fold higher infection rates with rituximab compared with ocrelizumab. Differences in patient characteristics, as well as in rituximab dosing regimens (typically repeated 1,000mg infusions biannually in the Cerono cohorts vs a single 500mg infusion biannually in Sweden), may partly explain these discrepancies.

However, reported rates of hospital‐treated infections with ocrelizumab in the 2 California cohorts were 4.4 and 8.4 per 1,000 patient‐years (PY), respectively; lower than the rates observed here for non‐BCDT DMTs,^1^ and comparable to the 6.75 per 1,000 PY rate reported in MS‐free controls.^11^ This contrasts markedly with their reported rates for rituximab, which ranged from 17.1 to 52.2 per 1,000 PY. Moreover, a pronounced difference in infection risk between ocrelizumab and rituximab is difficult to reconcile biologically, as both therapies deplete B cells with broadly similar repopulation kinetics.^12^, ^13^ We, therefore, consider it likely that the findings reported by Cerono and colleagues,^9^ despite ambitious efforts to control for confounding, may be influenced by systematic differences in patient selection as well as potential surveillance bias in outcome ascertainment, although differences in healthcare systems and treatment positioning of rituximab versus ocrelizumab may also contribute to variation in observed comparative risks across studies.

Strengths of the present study include access to a comprehensive dataset with detailed medical history, enabling adjustment for baseline differences, as well as its population‐based design. Limitations include the lack of primary care data (apart from prescription records) and the absence of information on factors such as body mass index, smoking status, and vaccination history. Residual confounding should, therefore, be considered when interpreting these findings, especially given the limited proportion of ocrelizumab‐treated patients, particularly among RRMS and treatment‐naïve patients. Further studies are needed to evaluate the potential impact of dosing intervals. Finally, we observed similar and substantially improved relapse control with both BCDTs compared with contemporary DMT comparators, with a particularly marked difference relative to cladribine. The comparable efficacy of rituximab and ocrelizumab has also recently been confirmed in a randomized head‐to‐head trial in newly diagnosed RRMS.^14^

In conclusion, we observe largely comparable infection risk profiles for ocrelizumab and rituximab, with only a higher rate of antibiotic use associated with ocrelizumab. Accordingly, our findings do not provide evidence to challenge the health‐economic advantages of using off‐label rituximab over ocrelizumab based on treatment‐related risks.^9^, ^15^

## Author Contributions

T.F. and F.P. contributed to the conception and design of the study; T.F., S.H., and F.P. contributed to the acquisition and analysis of data; TF and FP contributed to drafting the text or preparing the figures.

## Potential Conflicts of Interest

T.F. is partly funded by the non‐profit ARTIS project, which in turn is/has been supported by agreements between Karolinska Institutet and AbbVie, BMS, Eli Lilly, Galapagos, MSD, Pfizer, Roche, Samsung Bioepis, and Sanofi. S.H. has nothing to report. F.P. reports research grants from Denka, Janssen, Merck KGaA, Novartis, Pfizer, and UCB, and F.P. reports fees for serving on data monitoring committees in clinical trials with Lundbeck and Roche.

## Supporting information


**Table S1.** Definitions of outcome events.
**Table S2.** Definitions and categorization of potential confounding factors. Definitions and categorization of potential confounding factors.
**Table S3.** Censoring and reasons to discontinue treatment, by DMT group.
**Table S4.** Incidence rates, crude and adjusted hazard ratios from Cox regression, by individual DMT.
**Table S5.** Incidence rates, and crude and adjusted hazard ratios from Cox regression, by DMT among patients with Relapsing–remitting MS at DMT start.
**Table S6.** Incidence rates, crude and adjusted hazard ratios from Cox regression, for serious infection excluding COVID‐19.
**Table S7.** Inverse probability of treatment weighting as alternative way to control for confounding variables.
**Table S8.** Incidence rates, crude and adjusted hazard ratios from Cox regression, restricted to first ever DMT.

## Data Availability

Requests for sharing of de‐identified data will be considered on reasonable request and in accordance with current legislation regarding protection of sensitive personal data.
