# Supplementary material for: Comparable Infection Risk of Ocrelizumab and Rituximab in Multiple Sclerosis in a Nationwide Swedish Cohort Study
Source: Ann Neurol. 2026 Jun 17;100(4):788–92. doi: 10.1002/ana.78286 (PMC13587108; doi:10.1002/ana.78286)
Supplement: Supplementary file 1 [file ANA-100-788-s001.docx]

Online supplement for:

Comparable Infection Risk of Ocrelizumab and Rituximab in Multiple Sclerosis in a Nationwide Swedish Cohort Study

Thomas Frisell, PhD,^1^ Sebastian Hedberg, MSc,^1^ Fredrik Piehl MD/PhD^2,3^

1) Division of Clinical Epidemiology, Department of Medicine Solna, Karolinska Institutet, Stockholm, Sweden
2) Department of Clinical Neuroscience, Karolinska Institutet, Stockholm, Sweden
3) Academic Specialist Clinic, SLSO and Department of Neurology, Karolinska University Hospital, Stockholm, Sweden

Table of Contents

[Table S1. Definitions of outcome events	2](#_Toc226549651)

[Table S2. Definitions and categorization of potential confounding factors	3](#_Toc226549652)

[Medical history / comorbidity	4](#_Toc226549653)

[Table S3. Censoring and reasons to discontinue treatment, by DMT group	5](#_Toc226549654)

[Table S4. Incidence rates, crude and adjusted hazard ratios from Cox regression, by individual DMT	6](#_Toc226549655)

[Table S5. Incidence rates, and crude and adjusted hazard ratios from Cox regression, by DMT among patients with Relapsing-remitting MS at DMT start	7](#_Toc226549656)

[Table S6. Incidence rates, crude and adjusted hazard ratios from Cox regression, for serious infection excluding COVID-19	8](#_Toc226549657)

[Table S7. Inverse probability of treatment weighting as alternative way to control for confounding variables	9](#_Toc226549658)

[Table S8. Incidence rates, crude and adjusted hazard ratios from Cox regression, restricted to first ever DMT	10](#_Toc226549659)

## Table S1. Definitions of outcome events

| **Outcome** | **Codes** | **Data source** |
| --- | --- | --- |
| Serious infection | **ICD10:** A00-B99, D73.3, E06.0, E32.1, G00-G02, G04.2, G05-G07, H00.0, H44.0, H60.0-H60.3, H66-H67, H70, I30.1, I40.0, J00-J22, J32, J34.0, J36, J38.3, J39.0-J39.1, J44.0, J85, J86, K04.4, K04.6, K04.7, K10.2, K11.3, K12.2, K14.0, K57.0, K57.2, K57.4, K57.8, K61, K63.0, K65.0, K65.1, K65.2, K65.9, L00-L08, L30.3, M00-M01, M46.2-M46.5, M60.0, M65.0, M71.0, M71.1, M72.6, M86, N10, N11, N12, N13.6, N15.1, N15.9, N30.0 N30.8, N30.9, N34.0, N34.1, N34.2, N390, N41.2, N43.1, N45.2, N45.3, N45.4, N48.2, N61, N70, N73, N75, U07.1, U07.2. | Main diagnosis in the inpatient component of the Swedish National Patient Register |
| Infection | **ICD10:** A00-B99, D73.3, E06.0, E32.1, G00-G02, G04.2, G05-G07, H00.0, H44.0, H60.0-H60.3, H66-H67, H70, I30.1, I40.0, J00-J22, J32, J34.0, J36, J38.3, J39.0-J39.1, J44.0, J85, J86, K04.4, K04.6, K04.7, K10.2, K11.3, K12.2, K14.0, K57.0, K57.2, K57.4, K57.8, K61, K63.0, K65.0, K65.1, K65.2, K65.9, L00-L08, L30.3, M00-M01, M46.2-M46.5, M60.0, M65.0, M71.0, M71.1, M72.6, M86, N10, N11, N12, N13.6, N15.1, N15.9, N30.0 N30.8, N30.9, N34.0, N34.1, N34.2, N390, N41.2, N43.1, N45.2, N45.3, N45.4, N48.2, N61, N70, N73, N75, U07.1, U07.2. | Main or contributory diagnosis in the Swedish National Patient Register (in- and outpatient care at specialist/hospital clinics) |
| Antibiotics use | **ATC:** J01 | Any filled prescription in the Swedish Prescribed Drug Register |
| Relapses | Relapse recorded with a date | The Swedish MS Register |

## Table S2. Definitions and categorization of potential confounding factors

| **Variables** | **Categorization/Modelling** | **Data source** | **Time window for assessment at DMT start (days)** |
| --- | --- | --- | --- |
| **Year of treatment start** | Continuous | Swedish MS registry | Date of DMT start |
| **Age** | Continuous | Total Population Register | Date of DMT start |
| **Sex** | Female, male | Total Population Register | Not time varying |
| **Immigrant status** | Country of birth Sweden vs not-Sweden | Total Population Register | Not time varying |
| **Education** | ≤12 years or >12 years of education | LISA | Calendar year preceding the calendar year of DMT start |
| **Time since MS diagnosis** | Continuous  (Date of DMT start) | Swedish MS registry | Date of DMT start |
| **Nr prior DMTs** | 0, 1, 2+ | Swedish MS registry | Date of DMT start |
| **Any relapse within one year prior to inclusion DMT start** | Yes/no | Swedish MS registry | Lower: -365  Upper: 0 |
| **Expanded Disability Severity Scale (EDSS)** | Continuous | Swedish MS registry | Lower: -180  Upper: 30 |
| **Multiple Sclerosis Impact Scale-29 (MSIS-29) physical score** | Continuous | Swedish MS registry | Lower: -90  Upper: 30 |
| **Multiple Sclerosis Impact Scale-29 (MSIS-29) psychological score** | Continuous | Swedish MS registry | Lower: -90  Upper: 30 |
| **SDMT** | Continuous | Swedish MS registry | Lower: -90  Upper: 30 |

## Table S2 (continued). Definitions and categorization of potential confounding factors

| **Medical history** | **Codes** | **Data source** |
| --- | --- | --- |
| Serious infection | **ICD10:** A00-B99, D73.3, E06.0, E32.1, G00-G02, G04.2, G05-G07, H00.0, H44.0, H60.0-H60.3, H66-H67, H70, I30.1, I40.0, J00-J22, J32, J34.0, J36, J38.3, J39.0-J39.1, J44.0, J85, J86, K04.4, K04.6, K04.7, K10.2, K11.3, K12.2, K14.0, K57.0, K57.2, K57.4, K57.8, K61, K63.0, K65.0, K65.1, K65.2, K65.9, L00-L08, L30.3, M00-M01, M46.2-M46.5, M60.0, M65.0, M71.0, M71.1, M72.6, M86, N10, N11, N12, N13.6, N15.1, N15.9, N30.0 N30.8, N30.9, N34.0, N34.1, N34.2, N390, N41.2, N43.1, N45.2, N45.3, N45.4, N48.2, N61, N70, N73, N75, U07.1, U07.2. | Main diagnosis in the inpatient component of the Patient register |
| Infection | **ICD10:** A00-B99, D73.3, E06.0, E32.1, G00-G02, G04.2, G05-G07, H00.0, H44.0, H60.0-H60.3, H66-H67, H70, I30.1, I40.0, J00-J22, J32, J34.0, J36, J38.3, J39.0-J39.1, J44.0, J85, J86, K04.4, K04.6, K04.7, K10.2, K11.3, K12.2, K14.0, K57.0, K57.2, K57.4, K57.8, K61, K63.0, K65.0, K65.1, K65.2, K65.9, L00-L08, L30.3, M00-M01, M46.2-M46.5, M60.0, M65.0, M71.0, M71.1, M72.6, M86, N10, N11, N12, N13.6, N15.1, N15.9, N30.0 N30.8, N30.9, N34.0, N34.1, N34.2, N390, N41.2, N43.1, N45.2, N45.3, N45.4, N48.2, N61, N70, N73, N75, U07.1, U07.2. | Main or contributory diagnosis in the Patient register (in- and outpatient care) |
| Antibiotics use | **ATC:** J01 | Prescribed Drug Register |
| Malignancy | Any invasive malignancy (BEN≠3) | The Cancer Registry |
| Major Adverse Cardiovascular Event (MACE) | **ICD10:** I20.0, I21, I60, I61, I62, I63, I64 | Main or contributory diagnosis in the Patient register (in- and outpatient care) |
| Arrhythmia | **ICD10:** I44, I45, I47, I48, I49 | Main or contributory diagnosis in the Patient register (in- and outpatient care) |
| Antihypertensive treatment | **ATC:** C02, C03, C07, C08, C09 | Prescribed Drug Register |
| Diabetes mellitus | **ICD10:** E10-E14  **ATC:** A10 | Main or contributory diagnosis in the Patient register (in- and outpatient care)  Prescribed Drug Register |
| Antidepressant use | ATC: N06AB, N06AF, N06AX (except N06AX21) *(some antidepressants are excluded as they are commonly used to treat neuropathic pain in MS)* | Prescribed Drug Register |
| Baseline characteristics from the domain medical history was assessed with a series of binary variables, as presence vs absence of each condition in **the five years before treatment start**. Conditions are defined by ICD-10 coded diagnoses recorded in the Swedish National Patient Register and/or the ATC-codes for filled prescriptions in the Swedish Prescribed Drug Register. All codes in the table should read as “starting with”: A00 means all codes A00.0-A00.9ZZZ. | | |

## Table S3. Censoring and reasons to discontinue treatment, by DMT group

|  | RTX (n = 9704) | OCR (n = 148) | Other (n = 7020) |
| --- | --- | --- | --- |
| Censored | 7032 (72%) | 108 (73%) | 2036 (29%) |
| Discontinued treatment due to: |  |  |  |
| Anti-drug antibodies | 44 (0%) | 0 (0%) | 48 (1%) |
| Side effects | 463 (5%) | 9 (6%) | 1285 (18%) |
| Lack of effect | 208 (2%) | 9 (6%) | 1184 (17%) |
| Secondary progressive MS | 270 (3%) | 2 (1%) | 75 (1%) |
| Pregnancy | 86 (1%) | 0 (0%) | 237 (3%) |
| Planned pregnancy | 34 (0%) | 0 (0%) | 110 (2%) |
| Stable condition | 853 (9%) | 2 (1%) | 138 (2%) |
| Other reasons | 714 (7%) | 18 (12%) | 1907 (27%) |
| Notes: Censored means end of follow-up for another censoring condition than treatment discontinuation: emigration, death or (by far the most common) reaching the administrative end of follow-up Dec 31^st^ 2025. Abbreviations: OCR, ocrelizumab; RTX, rituximab. | | | |

## Table S4. Incidence rates, crude and adjusted hazard ratios from Cox regression, by individual DMT

|  | Person-years | Events | IR per 1000 PYs | HR (95% CI) | aHR (95% CI) |
| --- | --- | --- | --- | --- | --- |
| Serious infection |  |  |  |  |  |
| Rituximab | 48576.3 | 1048 | 22 | 1.0 (ref) | 1.0 (ref) |
| Ocrelizumab | 632.2 | 19 | 30 | 1.40 (0.89, 2.20) | 1.34 (0.81, 2.23) |
| Cladribine | 954 | 4 | 4 | 0.21 (0.08, 0.57) | 0.28 (0.10, 0.80) |
| Dimethyl fumarate | 11139.5 | 98 | 9 | 0.38 (0.31, 0.47) | 0.33 (0.23, 0.48) |
| Fingolimod | 3437.9 | 39 | 11 | 0.49 (0.36, 0.68) | 0.46 (0.36, 0.58) |
| Natalizumab | 6016.6 | 43 | 7 | 0.32 (0.24, 0.43) | 0.62 (0.43, 0.89) |
| Teriflunomide | 2735.9 | 31 | 11 | 0.50 (0.35, 0.72) | 0.58 (0.39, 0.85) |
| Infection |  |  |  |  |  |
| Rituximab | 42950.1 | 2779 | 65 | 1.0 (ref) | 1.0 (ref) |
| Ocrelizumab | 555.5 | 52 | 94 | 1.41 (1.07, 1.85) | 1.29 (0.95, 1.76) |
| Cladribine | 879.6 | 40 | 45 | 0.68 (0.50, 0.94) | 0.72 (0.51, 1.00) |
| Dimethyl fumarate | 10274.9 | 353 | 34 | 0.55 (0.49, 0.62) | 0.53 (0.44, 0.63) |
| Fingolimod | 3102.9 | 116 | 37 | 0.60 (0.50, 0.73) | 0.60 (0.52, 0.68) |
| Natalizumab | 5494.7 | 210 | 38 | 0.60 (0.52, 0.69) | 0.59 (0.47, 0.74) |
| Teriflunomide | 2544.3 | 93 | 37 | 0.58 (0.47, 0.71) | 0.66 (0.52, 0.83) |
| Antibiotics use |  |  |  |  |  |
| Rituximab | 27045.5 | 6016 | 222 | 1.0 (ref) | 1.0 (ref) |
| Ocrelizumab | 324.8 | 100 | 308 | 1.39 (1.14, 1.69) | 1.38 (1.10, 1.72) |
| Cladribine | 683 | 113 | 165 | 0.76 (0.63, 0.92) | 0.78 (0.63, 0.95) |
| Dimethyl fumarate | 6804.3 | 1224 | 180 | 0.73 (0.69, 0.78) | 0.92 (0.83, 1.02) |
| Fingolimod | 2040.7 | 330 | 162 | 0.67 (0.60, 0.75) | 0.78 (0.72, 0.85) |
| Natalizumab | 3631.7 | 754 | 208 | 0.85 (0.78, 0.91) | 0.68 (0.58, 0.79) |
| Teriflunomide | 1757.6 | 288 | 164 | 0.70 (0.62, 0.79) | 0.74 (0.64, 0.86) |
| Relapses |  |  |  |  |  |
| Rituximab | 50058.8 | 413 | 8 | 1.0 (ref) | 1.0 (ref) |
| Ocrelizumab | 670.5 | 6 | 9 | 1.23 (0.55, 2.75) | 0.99 (0.41, 2.40) |
| Cladribine | 885.8 | 32 | 36 | 5.59 (3.85, 8.11) | 6.31 (4.16, 9.58) |
| Dimethyl fumarate | 10973.5 | 286 | 26 | 2.24 (1.91, 2.62) | 1.50 (1.16, 1.94) |
| Fingolimod | 3401 | 65 | 19 | 1.69 (1.29, 2.21) | 3.05 (2.51, 3.72) |
| Natalizumab | 5858 | 112 | 19 | 1.79 (1.45, 2.21) | 2.03 (1.48, 2.78) |
| Teriflunomide | 2711.7 | 72 | 27 | 2.48 (1.93, 3.19) | 4.24 (3.16, 5.68) |
| Abbreviations: IR, incidence rate; PYs, person-years, HR, hazard ratio; CI, confidence interval. aHR, hazard ratio adjusted for age, sex, immigrant status, education level, MS course, number of previous DMTs, time since diagnosis, having experience relapse in last year, EDSS, MSIS-29, SDMT, and recent history of other disease, including infections, in the last five years. | | | | | |

## Table S5. Incidence rates, and crude and adjusted hazard ratios from Cox regression, by DMT among patients with Relapsing-remitting MS at DMT start

|  | Person-years | Events | IR per 1000 PYs | HR (95% CI) | aHR (95% CI) |
| --- | --- | --- | --- | --- | --- |
| Serious infection |  |  |  |  |  |
| Rituximab | 41548.6 | 742 | 18 | 2.28 (1.93, 2.68) | 2.19 (1.81, 2.64) |
| Ocrelizumab | 569.4 | 15 | 26 | 3.33 (1.96, 5.66) | 3.13 (1.71, 5.75) |
| Other | 23270.2 | 189 | 8 |  |  |
| Infection |  |  |  |  |  |
| Rituximab | 36415.9 | 2276 | 63 | 1.70 (1.57, 1.86) | 1.72 (1.55, 1.90) |
| Ocrelizumab | 493.9 | 46 | 93 | 2.48 (1.84, 3.35) | 2.38 (1.69, 3.35) |
| Other | 21361.0 | 762 | 36 |  |  |
| Antibiotics use |  |  |  |  |  |
| Rituximab | 23305.7 | 4977 | 214 | 1.30 (1.24, 1.37) | 1.25 (1.17, 1.33) |
| Ocrelizumab | 283.3 | 87 | 307 | 1.85 (1.49, 2.30) | 1.80 (1.41, 2.29) |
| Other | 14323.0 | 2547 | 178 |  |  |
| Relapses |  |  |  |  |  |
| Rituximab | 42364.2 | 365 | 9 | 0.49 (0.43, 0.56) | 0.39 (0.33, 0.46) |
| Ocrelizumab | 604.8 | 4 | 7 | 0.40 (0.15, 1.08) | 0.25 (0.09, 0.74) |
| Other | 22793.6 | 545 | 24 |  |  |
| Abbreviations: IR, incidence rate; PYs, person-years; HR, hazard ratio; CI, confidence interval. aHR, hazard ratio adjusted for age, sex, immigrant status, education level, MS course, number of previous DMTs, time since diagnosis, having experience relapse in last year, EDSS, MSIS-29, SDMT, and recent history of other disease, including infections, in the last five years. | | | | | |

## Table S6. Incidence rates, crude and adjusted hazard ratios from Cox regression, for serious infection excluding COVID-19

|  | Person-years | Events | IR per 1000 PYs | HR (95% CI) | aHR (95% CI) |
| --- | --- | --- | --- | --- | --- |
| Serious infection |  |  |  |  |  |
| Rituximab | 49375.8 | 825 | 17 | 2.32 (1.97, 2.72) | 1.83 (1.52, 2.21) |
| Ocrelizumab | 661.1 | 10 | 15 | 2.25 (1.19, 4.26) | 1.75 (0.88, 3.47) |
| Other | 24365.3 | 193 | 8 | 1.0 (ref) | 1.0 (ref) |
| Abbreviations: IR, incidence rate; PYs, person-years, HR, hazard ratio; CI, confidence interval. aHR, hazard ratio adjusted for age, sex, immigrant status, education level, MS course, number of previous DMTs, time since diagnosis, having experience relapse in last year, EDSS, MSIS-29, SDMT, and recent history of other disease, including infections, in the last five years. | | | | | |

## Table S7. Inverse probability of treatment weighting as alternative way to control for confounding variables

|  | Main analysis  aHR (95% CI) | IPTW  aHR (95% CI) |
| --- | --- | --- |
| Serious infection |  |  |
| Rituximab | 2.16 (1.82, 2.57) | 1.99 (1.68, 2.36) |
| Ocrelizumab | 2.92 (1.72, 4.98) | 2.51 (1.40, 4.52) |
| Other |  |  |
| Infection |  |  |
| Rituximab | 1.69 (1.53, 1.86) | 1.56 (1.42, 1.71) |
| Ocrelizumab | 2.18 (1.59, 3.01) | 1.82 (1.24, 2.69) |
| Other |  |  |
| Antibiotics use |  |  |
| Rituximab | 1.26 (1.18, 1.34) | 1.22 (1.15, 1.28) |
| Ocrelizumab | 1.73 (1.38, 2.17) | 1.67 (1.31, 2.13) |
| Other |  |  |
| Relapses |  |  |
| Rituximab | 0.38 (0.32, 0.44) | 0.48 (0.42, 0.56) |
| Ocrelizumab | 0.36 (0.15, 0.88) | 0.73 (0.24, 2.21) |
| Other | 1.0 (ref) | 1.0 (ref) |
| Abbreviations: IR, incidence rate; PYs, person-years, HR, hazard ratio; CI, confidence interval; IPTW, inverse probability of treatment weighting. aHR, hazard ratio adjusted for age, sex, immigrant status, education level, MS course, number of previous DMTs, time since diagnosis, having experience relapse in last year, EDSS, MSIS-29, SDMT, and recent history of other disease, including infections, in the last five years.  Notes: The main analysis (reproduced from Table 2) was a multivariable Cox regression, while the IPTW is a univariable but weighted Cox regression, where weights are the inverse of the propensity score, truncated to the 99^th^ percentile and stabilized by multiplication with the sample proportion with the specific DMT. The same variables and parameterizations were included in the two ways of adjusting for covariates. Balance (<0.1 standardized mean difference) was achieved for all covariates comparing the rituximab treated to the Other DMT group, but only for about half comparing ocrelizumab to rituximab. This lack of balance is not unexpected when so many factors are included in propensity score models for such a relatively small treatment group. | | |

## Table S8. Incidence rates, crude and adjusted hazard ratios from Cox regression, restricted to first ever DMT

|  | Person-years | Events | IR per 1000 PYs | HR (95% CI) | aHR (95% CI) |
| --- | --- | --- | --- | --- | --- |
| Serious infection |  |  |  |  |  |
| Rituximab | 16165.8 | 340 | 21 | 3.14 (2.42, 4.07) | 2.75 (2.01, 3.76) |
| Ocrelizumab | 149.9 | 4 | 27 | n/a | n/a |
| Other | 10122.8 | 70 | 7 | 1.0 (ref) | 1.0 (ref) |
| Infection |  |  |  |  |  |
| Rituximab | 14198 | 930 | 66 | 2.00 (1.75, 2.28) | 1.99 (1.69, 2.33) |
| Ocrelizumab | 138 | 9 | 65 | 1.96 (1.01, 3.81) | 1.70 (0.82, 3.53) |
| Other | 9430.4 | 303 | 32 | 1.0 (ref) | 1.0 (ref) |
| Antibiotics use |  |  |  |  |  |
| Rituximab | 9609.7 | 1920 | 200 | 1.35 (1.25, 1.45) | 1.31 (1.19, 1.45) |
| Ocrelizumab | 58.1 | 22 | 379 | 2.48 (1.62, 3.78) | 2.19 (1.30, 3.69) |
| Other | 6820.7 | 1081 | 158 | 1.0 (ref) | 1.0 (ref) |
| Relapses |  |  |  |  |  |
| Rituximab | 16640.2 | 113 | 7 | 0.39 (0.31, 0.49) | 0.32 (0.24, 0.43) |
| Ocrelizumab | 164.5 | 0 | 0 | n/a | n/a |
| Other | 9943.3 | 236 | 24 | 1.0 (ref) | 1.0 (ref) |
| Abbreviations: n/a, too few events; IR, incidence rate; PYs, person-years; HR, hazard ratio; CI, confidence interval; aHR, hazard ratio adjusted for age, sex, immigrant status, education level, MS course, number of previous DMTs, time since diagnosis, relapse in the past year, EDSS, MSIS-29, SDMT, and recent comorbidities, including infections, within the last five years. | | | | | |
